# Intrahepatic Cholestasis of Pregnancy: A Case Report of Third-Trimester Onset of the Disease

**DOI:** 10.7759/cureus.31926

**Published:** 2022-11-27

**Authors:** Hanadi A Bakhsh, Mamoun M Elawad, Reema S Alqahtani, Gharam A Alanazi, Maryam H Alharbi, Razan A Alahmari

**Affiliations:** 1 Department of Obstetrics and Gynecology, King Abdullah Bin Abdulaziz University Hospital, Princess Nourah Bint Abdulrahman University, Riyadh, SAU; 2 Department of Clinical Sciences, College of Medicine, Princess Nourah Bint Abdulrahman University, Riyadh, SAU; 3 Department of Clinical Sciences, College of Medicine, King Abdullah Bin Abdulaziz University Hospital, Princess Nourah Bint Abdulrahman University, Riyadh, SAU

**Keywords:** intrahepatic cholestasis, intrahepatic cholestasis of pregnancy, pruritus, third trimester, bile acids, pregnancy, cholestasis

## Abstract

Intrahepatic cholestasis of pregnancy (ICP) is characterized by pruritus and an elevation in serum bile acid concentrations, typically developing in the late second and/or the third trimester and rapidly resolving after delivery. ICP is a rare condition that most often presents in the late second or early third trimesters of pregnancy. Physicians should be aware of this unusual presentation of ICP and screen their patients appropriately during prenatal consultations by monitoring symptom severity and laboratory tests, including bile acids and liver enzyme levels, in addition to monitoring the fetal condition to start treatment modality including maternal treatment and determine the time of delivery to avoid fetal complications. This is a case of severe ICP diagnosed in the late second trimester and went into labor at 33 weeks of gestational age.

## Introduction

As a gestation-specific liver condition, intrahepatic cholestasis of pregnancy (ICP) is characterized by pruritus beginning in the third trimester of pregnancy, with elevated total serum bile acids (TSBA), and disappearing on its own after delivery. Its occurrence ranges from 0.1% to 15.6% worldwide, depending on location [1-3]. Between 2% and 5% of all pregnancies are affected globally [4]. Women who are pregnant for a third or more time or who are beyond the age of 35 years are at increased risk. When viral hepatitis is ruled out as a cause of jaundice in pregnancy, ICP is the next most common cause [5]. It has a complex etiology including interactions between genetic, endocrine, and environmental factors [6]. Although maternal outcomes are generally favorable, there does not appear to be an increased risk for postpartum hemorrhage when ICP is managed with ursodeoxycholic acid. However, fetal morbidity and mortality rise with ICP, including preterm birth, fetal discomfort, and abrupt intrauterine fetal death [7]. The most crucial step seems to be raising clinician knowledge of the possible negative fetal outcome of ICP and managing it as a high-risk pregnancy condition. A better prognosis for the unborn child may be achieved by timely and precise medical diagnosis and treatment [8]. This case study focuses on the effect of ICP on pregnant women and their newborns, including its diagnosis and treatment.

## Case presentation

**Figure 1:**
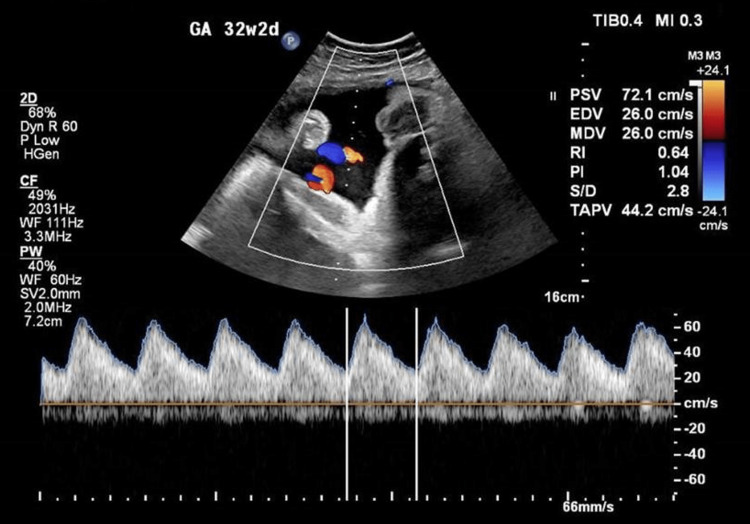
Antenatal ultrasound

A 33 weeks pregnant woman aged 30 years (G8 P0+7) was brought to the hospital at King Abdullah Bin Abdulaziz University because of severe, widespread pruritus that worsened at night and made it difficult for her to sleep. Laboratory tests verified the diagnosis, demonstrating a high rise of liver enzymes and total bile acids: complete blood count (CBC): hemoglobin (Hb) of 121 g/l (120-160). Liver enzymes and bile acids were elevated: alanine aminotransferase at 104 U/L (14-54), alkaline phosphatase at 102 U/L (32-91), lactate dehydrogenase at 153 U/L (98-192), and bile acids at 81.7 umol/l (6). Pruritus spread from the palms and soles to the rest of the body. After beginning treatment with ursodeoxycholic acid (250 mg) three times a day (TID), and performing weekly serum bile acids and fetal surveillance, including non-stress test (NST) and antenatal ultrasound, including biophysical profile and Doppler study (Figure 1), the patient's condition stabilized with stable lab values (CBC: Hb of 106 g/l) and normal platelets count. Liver enzymes and bile acids were elevated: alanine aminotransferase at 58 U/L (14-54), alkaline phosphatase at 312 U/L (32-91), lactate dehydrogenase at 202 U/L (98-192), and bile acids at 56.4 umol/l (6). She went into labor at 33 weeks, at which point she had to have a lower segment cesarean section due to the breech position of the baby. She gave birth to a healthy baby girl, who spent two days in the neonatal intensive care unit after being hospitalized for observation. As soon as the baby was born, the mother's symptoms went away. After giving birth, the patient no longer had any of her previous symptoms.

## Discussion

Symptoms of pruritus and increased bile acids are typical of intrahepatic cholestasis during pregnancy, a common disease. Due to a lack of evidence on the diagnosis, therapy, and accompanying adverse outcomes, the management has two main goals: reducing bothersome symptoms and reducing the risk of perinatal morbidity and mortality [9].

ICP is characterized by intense itching that begins between the 32nd and 36th week of pregnancy. One to four weeks after the development of pruritus, nausea, vomiting, and steatorrhea, jaundice may appear as a result of fat malabsorption [10]. Though all of these may be present in irritable bowel syndrome, chronic itching, particularly on the palms and soles of the feet, is the most common diagnostic symptom [11]. There is often a lag time of about three weeks between the start of pruritus and the rise of liver enzymes and blood bilirubin [12].

Women who develop intrahepatic cholestasis during pregnancy often have a history of biliary tract disorders such as gallstones, cholecystitis, cholangitis, and biliary tract cysts, either in their own lives or in their families [13]. It is more common in multiple gestations, chronic hepatitis C, prior history or family history of intrahepatic cholestasis, and advanced maternal age.

Roughly a third of patients indicate that their pruritus is at its worst on their palms and soles, before spreading elsewhere [9]. Nighttime, emotional stress, and increasing gestational age can make it worse. The itching often disappears within a week after giving birth [9,14]. Serum bile acid levels (40 mol/L), as reported by Glantz et al., are correlated with negative fetal outcomes [3]. Vitamin K deficiency, which causes bleeding during pregnancy or after childbirth, pruritus, and elevated bile acids and liver enzymes all go away within two days after giving birth. However, if these symptoms persist for more than a month, it may be a sign of chronic liver diseases such as primary biliary cirrhosis or chronic hepatitis [15].

The treatment's goals are to alleviate mothers' suffering, particularly from pruritus, and decrease perinatal death and morbidity. However, there are no standardized worldwide guidelines for measuring ICP in pregnant women. However, treatment hinges on keeping tabs on the severity of symptoms, bile acid levels, and fetal status.

Regular check-ins and diagnostic lab tests are a part of maternal monitoring (bile acids, alanine aminotransferase, aspartate aminotransferase, gamma-glutamyl transferase, bilirubin, and prothrombin time). Pregnancy monitoring should primarily use cardiotocography (CTG) and ultrasound screening, with intervals determined by the mother's pre-existing conditions and the total bile acid level [16].

Although there are drugs that may help with the symptoms of ICP, there is no proof that they enhance fetal outcomes. Ursodeoxycholic acid is a medication used to treat ICP, and it has been shown to alleviate maternal symptoms (particularly in women) and boost liver function. However, antihistamines or rifampin could be given in addition. Maternal blood bile acid levels are an important predictor of stillbirth and newborn problems, and hence should factor into the optimal delivery timing, which is patient-specific. Induction of labor is advised between 37 and 39 weeks of pregnancy, depending on the patient's bile acid level. If the bile acid level is below 100 mol/L, induction of labor is indicated at 36 weeks and zero days gestation. Fetal vitals should be monitored continuously during the labor process. Patients with ICP will have perfectly normal laboratory findings and clinical symptoms after giving birth [16].

## Conclusions

ICP poses a significant risk to the health of both the mother and the unborn child. Because the case is new in terms of diagnosis in the geographical area and because the treating institution is secondary care and the specificity of the case as it has a history of previous miscarriage, the researchers found the case to be inspiring and interesting. In this instance, the patient presented with significant ICP in the later stages of pregnancy and proceeded into labor at 33 weeks. While ICP may occur at any time during pregnancy, it often manifests in the late second or early third trimester. To start treating their patients as early as possible and avoid fatal complications, doctors need to be aware of this uncommon presentation of ICP and screen their patients appropriately during prenatal consultations by monitoring the severity of symptoms and laboratory tests, such as bile acids and liver enzyme levels.
